# Order and Disorder during *Escherichia coli* Divergence

**DOI:** 10.1371/journal.pgen.1000335

**Published:** 2009-01-23

**Authors:** Heather Hendrickson

**Affiliations:** Microbiology Unit, Department of Biochemistry, University of Oxford, Oxford, United Kingdom; Universidad de Sevilla, Spain

“… I was much struck how entirely vague and arbitrary is the distinction between species and varieties” —Charles Darwin, On the Origin of Species (1859)

*Escherichia coli* is a single species with numerous recognized roles, from lab workhorse to beneficial intestinal commensal or deadly pathogen. The extant strains have disparate lifestyles as a result of differential niche expansion since their divergence 25–40 million years ago, ten times longer than the estimated divergence between chimpanzees and humans [1],[2]. Not only do these roles vary by strain (variant) of the species, but the recognition of a strain's role in one context does not exclude radically different behaviour in another, due to differential gene expression [3]. These are organisms adapting on evolutionary and lifetime scales to myriad environments and pressures. How do these strains differ from one another and what sustains their identification as a single species?

To address these questions, Touchon et al. have completely sequenced and annotated six strains of *E. coli* while re-annotating previously sequenced strains, as discussed in this issue of *PLoS Genetics* [4]. Comparative genomics analyses of 20 *E. coli* strains and one out-group provided insights into the contributions of horizontal gene transfer (HGT) and mutation on evolution in this species. In addition, the strains were tested in a mouse model to compare their virulence.

## How Many Genes Could an *E. coli* Possibly Have?

It was known as early as 2001 that over 30% of the genes in *E. coli* O157∶H7 Sakai, a dangerous pathogen, are unique to that organism, compared with the K12 laboratory strain [5]. With the expansion of the data (from two genomes to 20) carried out by Touchon et al., the stark nature of the potential for similarities and differences between strains is revealed. The regions that are similar, the 4.1-Megabase “backbone” of the genomes, are 98.3% identical at the sequence level. This is remarkable considering the time they have had to diverge. Outside of this backbone, genes are in flux as a result of HGT and deletion. If expressed, genes gained through HGT can provide entirely new capabilities for a bacterium, ranging from carbon utilization to toxicity [6].

**Figure 1:**
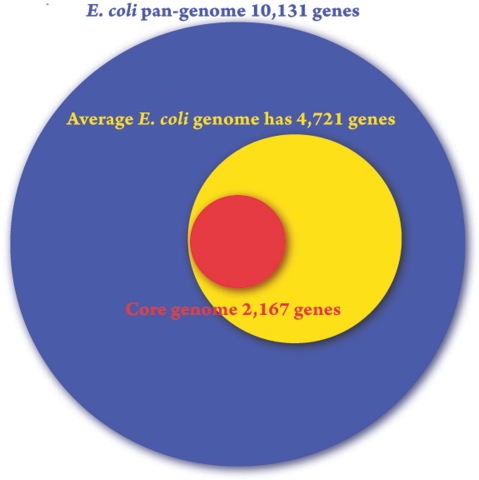
The breadth of genomic potential for *E. coli*. A Venn diagram of the pan-genome (blue), average genome (yellow), and core genome (red) of the sequenced *E. coli* strains according to Touchon et al. [4].

The collection of all genes found in the *E. coli* strains sampled is called the “pan genome” (Figure 1). Touchon and colleagues have found the *E. coli* repertoire to be a staggering 17,838 genes. Individual strains have an average of 4,721 genes, and it is estimated that only 1,976 of these will be the “core genes” that (nearly) all *E. coli* strains have.

## Consequences of a Large Pan Genome

Touchon et al. observe “… although some fundamental functions can be well studied by using a model strain, no single strain can be regarded as highly representative of the species” [4]. At first glance, this may seem a small point, but it calls into question a basic tenet of the International Code of Nomenclature of Bacteria, which still relies on the establishment of a “type species” that should not be “exceptional, including species which possess characters stated in the generic description as rare or unusual” (recommendation 20d.4). According to the authors, every *E. coli* strain is endowed with unusual characters, at least in terms of its gene content.

Large-scale genomic comparisons within a single species, particularly one with the range of lifestyles present in *E. coli*, have not been undertaken. Is *E. coli* atypical in terms of its catalogue of potential genes, or is it entirely normal? If such diversity continues to be observed at the single-species level, then we need to think carefully about what is meant by bacterial taxonomy [7].

## Signs of Selection

In addition to showing how these *E. coli* strains are different, the authors elucidate what makes them similar. The aligned core genome (those genes shared by a majority of the strains studied) was analyzed for linkage disequilibrium. Touchon et al. noted evidence for a high level of gene conversion: any single nucleotide was 100 times more likely to be involved in a gene conversion than a mutation. Mutation causes gradual change in DNA sequence, whereas homologous recombination restores similarity.

Even though a huge flux of HGT was observed, entrance of new DNA across strains was not random. In the 21 genomes analyzed, 133 locations were found to accumulate 71% of all the non-core pan-genome genes. For the majority of these, the participation of phage or integrase was ruled out. The formation of such hotspots for genomic flux cannot be explained by any known mechanism. Touchon et al. suggest that, once a rare, large integration event disrupts chromosome order, perhaps this less perfectly adapted region opens the way to future events through a “founder effect” for additional HGT. Phylogenetic incongruence tests revealed two chromosomal regions that were recombination hotspots with large selective footprints, indicating that this variation was being maintained by selection: the *rfb* and *leuX-fimH* loci. This is in agreement with other studies in both *E. coli* and *Salmonella enterica* [8],[9].

The terminus regions were found to have lower G+C percent contents than the rest of the genome, as well as a reduced ration of non-synonymous-to-synonymous polymorphisms and lower recombination rate. We may have much to learn about this region of the chromosome, potentially another example of the conflict between genomic flux and genomic organization.

## A Species by Any Other Name

Is the nature of melange-like *E. coli* truly captured by referring to its variants as individual strains? Far from a simple semantic argument, we need new concepts in evolutionary microbiology to refer to and understand organisms possessed of truly chimeric chromosomes. Even as we grapple to understand the breadth of present-day *E. coli*, they continue to evolve at a breathtaking rate. Since the first detection of *E. coli* 0157∶H7 in 1982, new sub-populations have emerged that have the capacity to cause even more serious illnesses [10]. Comparative genomics of the sort done by Touchon and co-authors unveils the complex evolutionary events taking place within these dynamic bacterial populations.
